# How useful are systematic reviews for informing palliative care practice? Survey of 25 Cochrane systematic reviews

**DOI:** 10.1186/1472-684X-7-13

**Published:** 2008-08-20

**Authors:** Bee Wee, Gina Hadley, Sheena Derry

**Affiliations:** 1 Sir Michael Sobell House, Oxford Radcliffe Hospitals, Churchill Hospital, Headington, Oxford, OX3 7LJ, UK; 2 Harris Manchester College, University of Oxford, Mansfield Road, Oxford OX1 3TD, UK; 3 Pain Research, Nuffield Department of Anaesthetics, University of Oxford, Churchill Hospital, Headington, Oxford, OX3 7LJ, UK

## Abstract

**Background:**

In contemporary medical research, randomised controlled trials are seen as the gold standard for establishing treatment effects where it is ethical and practical to conduct them. In palliative care such trials are often impractical, unethical, or extremely difficult, with multiple methodological problems. We review the utility of Cochrane reviews in informing palliative care practice.

**Methods:**

Published reviews in palliative care registered with the Cochrane Pain, Palliative and Supportive Care Group as of December 2007 were obtained from the Cochrane Database of Systematic Reviews, issue 1, 2008. We reviewed the quality and quantity of primary studies available for each review, assessed the quality of the review process, and judged the strength of the evidence presented. There was no prior intention to perform any statistical analyses.

**Results:**

25 published systematic reviews were identified. Numbers of included trials ranged from none to 54. Within each review, included trials were heterogeneous with respect to patients, interventions, and outcomes, and the number of patients contributing to any single analysis was generally much lower than the total included in the review. A variety of tools were used to assess trial quality; seven reviews did not use this information to exclude low quality studies, weight analyses, or perform sensitivity analysis for effect of low quality. Authors indicated that there were frequently major problems with the primary studies, individually or in aggregate. Our judgment was that the reviewing process was generally good in these reviews, and that conclusions were limited by the number, size, quality and validity of the primary studies.

We judged the evidence about 23 of the 25 interventions to be weak. Two reviews had stronger evidence, but with limitations due to methodological heterogeneity or definition of outcomes. No review provided strong evidence of no effect.

**Conclusion:**

Cochrane reviews in palliative care are well performed, but fail to provide good evidence for clinical practice because the primary studies are few in number, small, clinically heterogeneous, and of poor quality and external validity. They are useful in highlighting the weakness of the evidence base and problems in performing trials in palliative care.

## Background

In contemporary medical research, randomised controlled trials are seen as the gold standard for establishing treatment effects where it is ethical and practical to conduct them. In palliative care, randomised controlled trials may be impractical, unethical, or extremely difficult, with multiple methodological problems. The fact and nature of these issues with palliative care trials has been frequently commented upon [1-3]. Frequently encountered problems include recruitment and attrition, insufficient numbers of patients for any comparison, clinical heterogeneity between patients (condition palliated, comorbidity), heterogeneity in treatments (intervention, dose, duration), different outcomes reported, and use of non-standard scales. A palliative care Outcomes Working Group has recently made recommendations on outcomes they consider to be important in this context and how they might be sought in clinical trials [4].

Trials that have been done in palliative care are often small, diverse in nature and outcomes, and with high attrition rates, making meta-analysis, and even qualitative systematic review, impractical, unsatisfactory, or both. Moreover, some aspects of palliative care are difficult to capture, given the nature of palliative care as a person-centred approach, in which individual packages of care are often the norm [5].

With this background, the value of systematic reviews of randomized trials in palliative care might be questioned. One side of the argument would be that without a sufficiency of trials satisfying criteria of quality, validity, and size [6] systematic reviews are worthless. Another would see systematic reviews as a necessary first step to obtaining more evidence; despite their limitations, they at least tell us what we don't know, and may indicate how to improve.

This review set out to examine a subset of Cochrane reviews published under the auspices of the Pain, Palliative, and Supportive Care Review Group, to ascertain the number of successfully completed palliative care systematic reviews from this source over the last nine years, to assess their quality and the strength of the evidence presented to guide clinical practice.

## Methods

A list of published reviews relating to palliative care and registered with the Cochrane Pain, Palliative and Supportive Care Group was obtained from the Review Group Coordinator as of December 2007. Copies of each review were obtained from the Cochrane Database of Systematic Reviews using the most recent upload, issue 1, 2008.

The following information was extracted from each review:

• Number of studies included

• Number of patients included

• Condition palliated

• Intervention

• Trial design (randomized, observational)

• Measures of quality and/or validity used

• Whether exclusions due to poor quality were made, or a sensitivity analysis presented

• Whether a pooled analysis was done

• Original authors' conclusion on efficacy

• Original authors' conclusion on strength of evidence

• Original authors' implications for future research.

Two reviewers (GH, SD) independently carried out data extraction, using a standard form, and assessed the quality of each review using the Oxman & Guyatt Index of Scientific Quality [7]. To determine the strength of the evidence presented, for each review we assessed the quality of the included studies, based on randomization and blinding since these characteristics are known to affect potential bias [8], and the number of patients available for any analysis, because small numbers are prone to random error [9,10]. Any discrepancies were resolved by consensus.

There was no prior intention to perform any statistical analyses. What was intended was an evaluation of this set of systematic reviews in palliative care based on the quantity and quality of primary studies available, and the quality of the review process itself, in order to determine their utility for informing clinical practice.

## Results

Details of the 25 published systematic reviews [11-35] are in Additional file 1, together with the conclusions of the original authors. The first of these Cochrane reviews was published in 1999, and the most recent in 2007. Sixteen of the reviews concerned drug interventions for pain or other reasons, three involved radiotherapy, three complementary therapy, and one each for a mineral supplement, supportive care, and pleurodesis. Only five of the studies were published before 2003, and the rate of publication was five per year since 2004.

### Primary studies

#### Number of trials and patients

The numbers of included trials ranged from none to 54. Thirteen had fewer than five controlled trials, and 16 had fewer than 10 trials. Three reviews had between 11 and 20 trials, and six more than 20 trials. Six reviews had information on fewer than 100 patients in total in controlled trials, fourteen had fewer than 500, while eight had between 1000 and 5000, and one more than 6,000 patients (see Additional file 1: Included reviews). Within each review, included trials were frequently heterogeneous, with differing interventions (drug, dose, route, technique) and reported outcomes, so that the number of patients contributing to any single analysis was nearly always much lower than the total number of patients included in the review.

All the reviews sought randomised controlled trials for inclusion. Five reviews [22,25,26,30,34] sought uncontrolled studies, but only two analysed these in the absence of randomised trials [22,26]. Two reviews [28,31] found no studies that met their inclusion criteria.

#### Types of patients

Eighteen reviews included trials involving only cancer patients. In most cases the type of cancer or site of the primary cancer was not restricted. One review included only AIDS patients [27], two included mixed diagnoses of cancer, lung disease, cardiac failure, cystic fibrosis, and elderly patients [20,33], and one included patients with cancer or unspecified "terminal illness" [14].

### Original authors' assessment of quality of included studies

All reviews with included studies assessed their quality, with the exception of Ballantyne [22] and Quigley [26], who found no randomised trials and included mainly retrospective studies, audits, or case reports, and uncontrolled prospective cohort studies. A number of scales were used. Most (18/23) used the Oxford Quality Score [8], and of these, three [16,29,30] additionally used the Oxford Pain Validity Scale [36], two [14,21] used Rinck [1], one [17] used Detsky [37], and another [15] used both Juni [38] and Delphi [39]. Shaw [24] graded trials according to criteria in the Cochrane Handbook [40], Feuer [34] according to Mann [41], and Ezzo et al [18] used their own set of five questions. Seventeen reviews also assessed allocation concealment using Cochrane criteria [42] in at least some of the included trials. Eight of the reviews that assessed trial quality did not use the information to exclude low quality studies, weight analyses, or perform sensitivity analysis for effect of low quality [11-15,23-25]. For details of quality scores of included studies see Additional file 2 (Adequacy of included studies), and of the quality scoring tools used see Additional file 3 (Quality and validity tools).

The original authors themselves indicated that there were frequently major problems with the primary studies, individually or in aggregate. These included low numbers (either in total or available for pooled analysis) in 18 cases, the lack of useful outcomes in 10, methodological heterogeneity in eight, design problems in five, and clinical heterogeneity in two. For example, one review stated that we " ... need more larger studies with standardised outcomes of clinical relevance and clearer definitions of best supportive care" [21], while another stated that "Trials were too ...... short term for results to be meaningful" and that "Clinically relevant questions to address include which compounds are most beneficial, optimal dose and administration route, when prophylactic therapy ... should be started ..." [29].

### Reviewers' assessment of quality of reviews

The methods used in these 25 reviews appeared to be sound. We attempted to use the Oxman & Guyatt Index of Scientific Quality [7], which asks questions about review methods. All the reviews had effective search strategies, and all looked at methodological quality in some way. However, deficiencies in the primary studies made judgment about assessment of validity and combining data close to impossible, as it was for the original authors. For instance, many reviews made no attempt to combine studies in a pooled analysis because of clinical heterogeneity and diverse interventions and outcomes, a decision that we felt to be correct.

We also felt that an overall Oxman & Guyatt score for these reviews was inappropriate because it attempts to measure flaws in the reviewing process. Our judgment was that the reviewing process was generally good in these reviews. Limited amounts and quality of data limited conclusions about efficacy or harm, most importantly lack of patient numbers, poor/inconsistent reporting, frequent use of non-standard outcome measures, and excluding outcomes which lack clinical relevance, for example patient satisfaction and long-term morbidity.

In our assessment of the strength of the evidence presented, we found that of the 25 reviews:

• 2 had no data – there were no trials found [28,31];

• 2 included uncontrolled trials [22,26], known to be the subject of significant bias [8];

• 12 included randomized trials, but with open or non-blinded designs [12,14-17,19-21,23-25,35], again known to be the subject of bias, especially in pain [43];

• 4 included randomized trials, with a mix of blind and open designs. Of these:

○ Wong [32] included mostly double blind studies, with 3600 patients, but using different drugs, doses, and routes of administration;

○ Nicholson [11] had 460 patients and 6/9 trials were double blind, but with different doses, and routes of methadone administration, and different comparators;

○ Dewey [13] had 60 patients and 4/5 trials were double blind, but they were insufficiently rigorous to be confident of any effect;

○ Ezzo [18] had 1250 patients in acupuncture trials, with a mix of techniques and controls. The trials and review have been criticized elsewhere [44];

• 5 included randomised trials with only double blind design. Of these:

○ Three had fewer than 100 patients [27,30,34];

○ Roque [29] had only 325 patients in 4 trials using different drugs, and doses, in single or multiple dose schedules, and for different duration;

○ Jennings [33] had 292 patients in 3 trials, but with different drugs and doses.

Two reviews [20,35] were considered to have the strongest evidence, although even for these reviews there were limitations with methodological heterogeneity or definition of outcomes. No review provided strong evidence of no effect. Even reviews with relatively large numbers of trials and patients could not provide strong evidence because of inappropriate comparison or trial design [23] or methodological heterogeneity [17].

## Discussion

This systematic review of systematic reviews in palliative care was to question the utility of systematic reviews for informing clinical practice in this area of medicine. It found that 25 reviews were published in the Cochrane Database of Systematic Reviews over nine years, a rate of about 2.7 per year overall, though almost double that rate occurred in the three years to 2007. Despite a respectable level of productivity from this prestigious source, 22/25 reviews could produce only weak evidence of the benefits of any intervention, and even of the two where the evidence was considered to be strong there were caveats.

The review processes themselves appeared adequate. Deficiencies lay in the primary studies, which were either missing or scant, or were characterized by heterogeneity in the methods, interventions, patients, and outcomes, which made an overall assessment of benefit or harm impossible. These deficiencies are similar to those identified previously [1-3,5]. The authors of the reviews commonly commented on these deficiencies, and others. The biggest single issue was that of inadequate trials or inadequate patient numbers in high quality trials. In making even this point, the reviews and the reviewers make an important contribution.

It is likely that these observations are general to systematic reviews in palliative care. We limited our investigation to reviews from the Cochrane Database published through the auspices of the Palliative Care group, but we would expect such reviews to be no worse, and perhaps better, than non-Cochrane reviews [45,46]. The restriction to Cochrane reviews should not limit any generalisability of these findings, especially as this reasonably sized body of reviews consistently makes the same, or very similar, points.

These findings are not a surprise. The dearth of good quality primary studies in the field of palliative care is widely accepted, and those trials that have been done are often known to have weaknesses [1-3,5]. Together, these factors underline the limitations of the knowledge base upon which palliative care has to draw. Whether new guidance about outcomes to be measured in palliative care trials would make a difference [4] remains to be seen, but given the difficulties in design and conduct of palliative care trials, rapid change in the corpus of evidence is unlikely.

The challenge for palliative care is the lack of evidence that is available to support it and the inordinate difficulties in obtaining evidence, for example difficulties with recruitment and attrition in an ill and vulnerable population. This has led to calls for a different framework for examining evidence [47]. Part of the problem is that nearly all randomised controlled trials examine single interventions, while in clinical practice that intervention will often form a small part of a much larger overall package of care [5]. Randomised trials of overall packages of care with small or incremental differences between them are unlikely to be able to measure small improvements, and an evaluation of systematic reviews of palliative care services [48] highlighted similar problems to those of palliative care interventions. High patient losses also make interpretation of randomised trials difficult. It is important that palliative care research moves away from dependence on randomized trials, and explores alternative study designs to identify the most effective treatments and packages of care for its patients.

There may be alternatives. Nearly all the Cochrane reviews included only randomised trials, and the small number of reviews that did consider non-randomised studies found them to have many of the same problems as randomised trials, with an additional increased risk of bias. We know, from other areas of medicine, that high quality, well-formulated, and impeccably conducted large observational studies, can provide equivalent results to those obtained from randomised controlled trials [6,49,50]. To overcome the play of chance these good quality studies need to be large, and to minimise bias they need to be both prospective and inclusive (i.e. a whole population, or all patients attending a clinic in a defined time). Registry studies are studies based on information from registers that systematically record information from all individuals in a defined population. They can be entire populations, as in the death register in the UK, or all patients with a specific characteristic (eg twins) or condition (eg breast cancer) within a defined population. At least one large registry-based programme for continuous quality improvement aimed at cancer pain is ongoing in Italy [51]. An extensive search for observational studies in palliative care has been undertaken, with the aim of identifying good quality observational studies and aspects of their design that make them reliable and useful (Hadley et al., manuscript in preparation). The proven limitation of controlled trials in palliative care may make registry studies a more acceptable option in future.

## Conclusion

Cochrane reviews in palliative care are well performed, but fail to provide good evidence to guide clinical practice because the primary studies are few in number, small, clinically heterogeneous, and of poor quality and external validity. These reviews do, however, tell us how limited the evidence base is, and highlight common deficiencies in primary studies. There are well-documented problems with conducting valid randomised trials in this area, and it may be that for some questions more, and more clinically relevant, information can be obtained from other types of primary study, such as large registry studies.

## Competing interests

BW and SD have received research support from charities, government and industry sources at various times, but no such support was received for this work. No author has any direct stock holding in any pharmaceutical company.

## Authors' contributions

GH and SD were involved with planning the study, data extraction, analysis, and preparation of the manuscript. BW was involved with planning the study and preparation of the manuscript. All authors read and approved the final manuscript.

## Pre-publication history

The pre-publication history for this paper can be accessed here:



## Supplementary Material

Additional file 1 Included reviews. Details of included reviews. Click here for file

Additional file 2 Adequacy of included studies. Details of design, size, and quality of primary studies in each review. Click here for file

Additional file 3 Quality and validity tools. Details of tools used to assess quality and validity in primary studies. Click here for file
