# Supplementary material for: How useful are systematic reviews for informing palliative care practice? Survey of 25 Cochrane systematic reviews
Source: BMC Palliat Care. 2008 Aug 20;7:13. doi: 10.1186/1472-684X-7-13 (PMC2532992; doi:10.1186/1472-684X-7-13)
Supplement: Additional file 1 — Included reviews. Details of included reviews. [file 1472-684X-7-13-S1.pdf]

Additional file 1: Included reviews

Reference	Condition palliated	Included studies	Max number of patients	Intervention	Original authors' conclusion	Authors' comment on strength of evidence	Author's implications for future research
**Nicholson (2007;i4)** Methadone for cancer pain	Cancer pain of any intensity, in any setting	9 trials: all R, C, and 6 DB	459	Any dose (single or multiple), any route, placebo or different active comparators	Methadone has a similar efficacy to morphine in treating cancer pain, and is no more effective than morphine for cancer-related nerve related pain. Its side effect profile is similar to morphine, but these side effects may become more prominent with repeated dosing.	Conclusions limited by variations in trial design, dosing regimens and limited presentation of primary outcome data. Only one trial attempts to mimic use of methodone in clinical practice (regular dosing). Insufficient data on different pain syndromes to show differences	Repeated dose studies using methadone at fixed dose intervals is potentially hazardous, making double blind trial against an alternative opioid given regularly almost impossible. Randomization to placebo or methadone for patients with cancer pain is unacceptable to most clinicians. Methodology of future studies should address issues of dose titration schedules, use of standard and comparable pain intensity scores, use of patient reported outcomes only, study size and differentialtion of pain syndromes, measures of patient satisfaction and quality of life
**Wiffen (2007;i4)** Oral morphine for cancer pain	Moderate/severe cancer pain	54 trials: all R, C Max 17 trials in any comparison	3749 Max 973 in any comparison	Single/multiple dose, different formulations, different doses, different comparators	Oral morphine is an effective analgesic for some patients with cancer pain. Titration to pain relief is possible using modified release morphine. Adverse effects common but often tolerable. Small number of patients do not benefit or develop intolerable adverse effects	Qulaity of tirals diappointing. Trials designed to show equivalence not effectiveness. Many small, crossover design without adequate washout, inconsistent reporting of adverse event withdrawals	Need to assess pain and pain relief by means of patient-reported validated scales, and to present data that can be related to individual subjects rather than aggregated or mean data
**Dewey (2007:i1)** Eicosapentaenoic acid (EPA, an omega-3 fatty acid from fish oils) for the treatment of cancer cachexia	Cancer cachexia	5 trials: all R, C, 4 DB	587	Oral fish oil dietary supplement	There is is insufficient evidence to recommend the use of EPA in clinical practice. There is little evidence of harm	There is a paucity of well-conduceted RCTs to answer the review question	There is a need to conduct good quality, large scale RCTs using EPA compared to placebo with different cancer types. Issues around clinical stage at recruitment, clinically important outcomes, use of other supportive therapies, and palatability of EPA need to be addressed
**Miles (2006;i4)** Laxatives for the management of constipation in palliative care patients	Cancer	3 trials: all R, open	163	Five different laxatives (or combinations), different doses	All treatments had limited efficacy (ineffective for significant numbers of patients). Unable to distinguish between treatments	Too few comparative studies of laxatives or combinations of laxatives to determine”best” treatment for constipation	Important to consider degenerative neurological disease, AIDS, end stage organ system failure, dementia and chronic lung disease alongside cancer in the palliative care setting. Randomised, controlled trials in clearly defined populations, measuring standardized, clinically relevant outcomes are required. Need comparisons of efficacy of combination laxatives against single laxatives. Different physical attributes of available laxatives make blinding difficult. Multi-centre studies could help combat high attrition rates and small numbers of eligible patients

**Dennert (2006;i3)**<br>Selenium for alleviating the side effects of chemotherapy, radiotherapy and surgery in cancer patients	Malignant disease treated with tumour specific therapy (chemo, radiotherapy, surgery)<br>Any stage of disease, adults and children	2 trials: both R, C (one presenting preliminary results)	123	Inorganic selenium supplements, diff dosage regimens, different durations	There is insufficient evidence to recommend selenium supplements to relieve side effects of chemotherapy, radiotherapy or surgery, or improve QoL in cancer patients	Lack of primary data contrasts with number of secondary publications recommending use, and proportion of cancer patients using selenium supplements	Adequate dosage-finding study required, with adequate reporting of completed, ongoing and future trials
**Martinez-Zapata (2006;i3)**<br>Calcitonin for metastatic bone pain	Metastatic bone pain (both trials in breast cancer patients)	2 trials: both R, C	90	Drug, dose and route consistent, but duration different	Limited evidence does not support the use of calcitonin to control pain from metastases	No data for clinically useful outcomes other than pain	Need more double blind, parallel clinical trials using long-term evaluations, with realistic sample size calculations, including adequate estimations of number of patients likely to be lost to follow-up.<br>Alternatively, variables studied should be confined to reduction/absence of pain, adverse effects, and QOL. Possible complications of bone metastases such as hypercalcemia, bone fractures or radicular compression, should also be quantified
**Tsao (2006;i3)**<br>Whole brain radiotherapy for the treatment of multiple brain metastases	Multiple metastases to the brain from any primary cancer	24 trials: various designs, not all full publications	6353	Different fractionation schedules (9); WBRT with vs without systemic therapy (5), various radiosensitisers (5) or radiosurgery (4); WBRT plus steroids vs steroids alone (1)	No additional benefit (survival, neurologic function or symptom control) over standard WBRT for altered dose schedules. Use of adjunctive chemotherapy or radiosensitisers still experimental	Lack of evidence for WBRT vs supportive care, and criteria to help select best treatment for different categories of patients	Future trials should examine use of whole brain radiotherapy versus supportive care alone in patients with multiple brain metastases. Clinically relevant outcomes should be defined, including quality of life, symptom control, neurological function, optimal timing of radiosurgery in relation to whole brain radiotherapy, steroid toxicity, and overall survival
**Ezzo (2006;i2)**<br>Acupuncture-point stimulation for chemotherapy-induced nausea or vomiting	Cancer patients with chemotherapy-induced nausea and/or vomiting	11 trials: all R, C	1247	Different stimulation techniques (manual and electroacupuncture, acupressure, non-invasive electrostimulation) and controls (sham, non-sham)<br>Therapy adjuctive to antiemetics - some outdated	Electroacupuncture seems to pretect against acute vomiting, and acupressure against acute nausea	Methodology of included trials mixed.<br>Studies needed in refractory patients and using modern antiemetics to determine clinical relevance of findings	Lack of sham control in some studies made it difficult to interpret nausea scores, a subjective outcome. Lack of concurrent modern antiemetics in electroacupuncture studies, makes it impossible to assess whether acupuncture can offer adjunctive benefit on top of modern antiemetics
**Zeppetella (2006;i1)**<br>Opioids for the management of breakthrough (episodic) pain in cancer patients	Breakthrough cancer pain, in any setting	4 trials: all R. C	393	Oral transmucosal fentanyl citrate (OTFC) dose titratiion (2), vs sr morphine (1), or vs placebo (1)	OTFC effective for control of breakthrough pain, and preferred to sr morphine. Dose needs to be determined by titration	Few, small trials, and no evidence for other opioids	Included studies confirm that randomised controlled studies are possible in palliative care setting. The randomised trial literature for the management of breakthrough pain is small and no trials were found for other opioids

**Berenstein (2005;i2**) Megestrol acetate for the treatment of anorexia-cachexia syndrome	Anorexia-cachexia due to cancer, AIDs or other pathologies (3445 cancer, 435 AIDs, 243 other)	30 trials: all R, C	4123 Only 50% contributed to analysis	Different doses, placebo and/or active control, dose response Different durations	Megestrol acetate improves appetite and weight gain in cancer patients. No evidence of dose response. No overall conclusion about QOL possible due to heterogeneity. Insufficient data for AIDs and other patients. Adverse event rate low	Limited by quality of primary studies, and number in non-cancer patients	Results of meta-analysis are influenced by the quality of primary studies included. Studies of low methodological quality can alter the interpretation of the benefit of intervention. In this review 64% of studies were assessed as moderate or low quality trials. Studies with more than 50% of patients lost to follow-up were excluded from the analysis. Quality of life is a subjective measure and such measures must be valid and reliable
**Ahmed (2005;i1)** Supportive care for patients with gastrointestinal cancer	Advanced, metastatic gastric or colotrectal cancer	4 trials: all R, C	483	Four different chemo regimens compared to different packages of supprtive care (2) or best supportive care (2)	Addition of chemotherapy to supportive care gives benefit in survival and quality of life. Insufficient evidence on pain or symptom control	Need more larger studies with standardised outcomes of clinical relevance, and clearer definitions of best supportive care	Need validated instruments that examine symptom control, quality of life, toxicity, pain severity and pain relief in addition to survival and other palliative measures, both before and after treatment completion. Upper age limits in trials do not reflect the age distribution of the disease. Need improved criteria for including supportive care interventions into cancer RCTs
**Ballantyne (2005;i1)** Comparative efficacy of epidural, subarachnoid, and intracerebroventricular opioids in patients with pain due to cancer	Intractable cancer pain	No controlled trials 72 uncontrolled trials	2402	Drug, dose, dosing schedule not considered - just route of administration	IVC is at least as effective as EPI or SC opioids for refractory cancer pain. Different routes associated with slightly different adverse event profiles	Uncontrolled trials can provide only weak evidence and may well be skewed by unbalance populations (confounding by indication)	Patients entering trials of ICV are varied and we may not be comparing equivalent populations. ICV population apparently had more advanced disease and worse pain. More rigorous reporting of efficacy and complications is required – multiple uncontrolled trials were reported on
**McNicol (2005;i1)** NSAIDS or paracetamol, alone or combined with opioids, for cancer pain	Cancer pain not limited to palliative setting	42: all R, C	3084	Different NSAIDs and combinations, different comparators, different doses, different routes of administration, single/multiple dosing, different durations	Limited data indicate that NSAIDs are more effective that placebo, but do not show differences between drugs. Combination with opioid shows no difference to slight benefit. Short duration of studies limits clinical relevance. WHO pain ladder step 2 is not supported by evidence	Conclusions limited by heterogeneity in studies and clinical relevance. Few trials contributed to any one subcategory of analysis	Heterogeneity of study designs prevented comprehensive meta-analysis and prevented definitive conclusions, despite large number of patients. Cancer pain generally chronic in nature so inappropriate to use methods for deriving dichotomous outcome measures from continuous data in acute pain studies (McQuay, 1998). Use of placebo for pain management in cancer trials may be unethical (Jacox 1994). Studies to ascertain whether addition of opioid to NSAID regimen actually increases efficacy and/or reduces side effects are required. Need to establish safety and efficacy of chronic NSAID use in patients with cancer
**Shaw (2005;i1)** Pleurodesis for malignant pleural effusions	Malignancy leading to pleural effusion	36 trials: all R, all open	1499	Many sclerosants used, predominantly bleomycin, tetracycline and talc. Bedside or thoracoscopic pleurodesis	Thoracoscopic pleurodesis with talc may be the optimal technique. No evidence for increased mortality with technique or individual sclerosant	Need standard definitions of success and failure, and more consistent reporting of adverse events, withdrawals etc. Need studies to determine best technique, timing and management of patients	Standardised criteria should be used for methods of assessing adverse events and toxicity and reporting procedure-related morbidity and mortality. Standardised criteria required to assess success or failure of pleurodesis and clinically relevant quality of life outcomes e.g. breathlessness, cough, discomfort and pain, fatigue, and reduced exercise capability

**Fellowes (2004;i3)** Aromatherapy and massage for symptom relief in patients with cancer	Cancer patients (adults and children) receiving health care in any setting	8 trials: all R, C	357	Massage vs no-massage control (6) and aromatherapy massage vs carrier oil massage (2) Duration of sessions, total treatment period, experience of masseur and nature of oils variable	Massage and aromatherapy massage confer short-term benefits on psychological wellbeing. Anxiety may be reduced, but no evidence for reduction in depression. Insufficient evidence for effect on physical symptoms	Methodological difficulties encountered, particularly of size and blinding Determination of most effective treatment regimen and longer-term outcomes needed	Need longer follow up studies to determine whether short-term effects persist, and larger sample sizes. Most advantageous number of massages, and areas of body to be massaged, using same outcome measures and scales, would also strengthen evidence base
**Quigley (2004;i3)** Opioid switching to improve pain relief and drug tolerability	Adults and children with chorinic or acute pain Not limited to palliative care setting	No RCTs 52 reports: case reports (23), retrospective studies/audits (15), prospective uncontrolled studies (14)	Not clear Prospective and retrospective studies ±2000 Case reports 55	Morphine generally first choice and methadone second. Conversion ratios varied or not reported Reason for switch often not reported	Robust evidence for the practice of opioid switching does not exist. Uncontrolled data suggest that for some patients it may improve pain or tolerability	Studies often small, and open to bias due to poor quality. Publication bias likely	Differences in opioid formulation make blinding difficult. Blinding of outcome assessors and patients should be possible, and 'N of 1' trial design. Opioids with similar pharmacokinetic profiles could be compared for toxicity and analgesia in RCTs. Information on which opioids are safe in patients with renal impairment would significantly improve management in these patients. Wide gaps in our understanding of inter-individual variability in opioid response, so need to test hypothesis that opioid switch is useful for some patients with uncontrolled pain and/or intolerable adverse effects
**Jackson (2004;i2)** Drug therapy for delirium in terminally ill patients	AIDs	1 trial: R, DB	30	Haloperidol, chlorpromazine and lorazepam. Dose according to recognised protocol	There is insuffiecient evidence to draw conclusions about the role of drugs for delirium in palliative care. Haloperidol and chlorpromazine may help, but the latter may cause cognitive impairment	Only one study with 30 patients. More research is needed	To date no randomized, placebo-controlled trials have been conducted in patients with advanced cancer or other terminal disease states. Generates important research questions
**Jackson (2004;i1)** Drug therapy for anxiety in palliative care		None			There is insufficient evidence to recommend any therapy for anxiety in palliative care		Main reasons for study exclusion were (a) patient populations not considered to be specifically terminally ill (b) studies not prospective and with a pharmacotherapeutic comparison. Generated some specific research questions for future research
**M Roqué (2003;i4)** Radioisotopes for metastatic bone pain	Metatstatic bone pain	4 trials: all R, DB, PC	325	Different drugs, different doses, single/multiple doses, different duration	Some evidence that radioisotopes may give complete pain relief in the short term, but with increased risk of serious adverse events. No effect on spinal cord compression. No data for long term	Trials were too small and too short-term for results to be meaningful. No data for other clinically useful oucomes	Need rigorous parallel, double blind clinical trials including long-term evaluations and larger sample sizes, with properly estimated losses to follow up due to mortality or disease progression. Clinically relevant questions to address include which compounds are most beneficial, optimal dose and administration route, when prophylactic therapy for bone complications should be started, identification of groups that benefit most from therapy, and cost-effectiveness of each compound

**Bell (2003;i3)** Ketamine as an adjuvant to opioids for cancer pain	Cancer pain	2 trials: both R, DB, cross-over, adjuvant therapy. 32 case reports or uncontrolled studies also reviewed	30	Ketamine, either iv or intrathecal. Different doses, one used washout, other did not	Insufficient evidence to assess ketamine as adjuvant to morphine	Need larger trials and to consider opioid tolerance, nature of pain, and route of administration. Need well defined, clinically relevant outcomes	Provides good framework for future studies. Difficult to recruit patients for trials could be addressed by crossover design, which may be more appropriate than placebo-controlled parallel studies. Need standardisation of doses and reported pain, large numbers of patients, and to address issue of possible effect of ketamine in prevention of opioid tolerance.Identification of clinically relevant outcomes is paramount i.e. effective doses and adverse events. In-house data bases from pharmaceutical companies may be an effective way of searching
**Hirst (2002;i4)** Benzodiazepines and related drugs for insomnia in palliative care	Pallaitive care	None			Extensive searching failed to identify any RCTs of benzodiazepines for insomnia in palliative care settings		Need large, good quality randomized controlled trials involving representative patients with incurable progressive medical conditions, with explicit subjective complaints of insomnia, of sufficient duration, and measurement of all relevant outcomes. Outcome measures should involve subjective evaluations of sleep quality and adequately monitor adverse effects
**Wong (2002;i2)** Bisphosphonates for the relief of pain secondary to bone metastases	Bony metatases from any primary neoplasm	30 trials: all R, 21 DB and PC, 4 open, 5 active (dose response)	3582	Etidronate (3), clodronate (15), pamidronate (12) Oral, iv or mixture Different doses	Bisphosphonates provide modest pain relief for patients with painful bony metastases	Data available for meta-analysis are so limited that no robust conclusions can be reached	Most important and clinically relevant endpoint for inclusion in quantitative reviews is proportion of patients with pain relief, described for each arm of the trial. Mean pain scores are not helpful
**Jennings (2001;i3)** Opioids for the palliation of breathlessness in terminal illness	Mainly COPD, some cancer, cardiac failure, lung disease Mostly outpatients	18 trials: all R, DB, PC, cross-over	292	Dihydrocodeine, diamorphine, morphine Different doses, formulations - oral, (standard, immediate, slow release), sc, iv, nebulised	Strong evidence for small, probably clinically significant effect of oral and parenteral opiods on breathlessness No evidence for benefit from nebulised opiods for breathlessness or any opiod for exercise tolerance	Limited by small numbers of patients, and lack of standardised outcomes and reporting	Need trials with larger numbers of patients, using standardised protocols. Variety of different outcome measures were used in breathlessness studies. Even within one measure (eg Borg score), outcome often reported differently (often not at fixed point relative to exercise). Quality of life measures needed. Problems of using different types of palliative care patients stem from fact that cancer patients are more heterogeneous than COPD patients
**Feuer (1999;i3)** Corticosteroids for the resolution of malignant bowel obstruction in advanced gynaecological and gastrointestinal cancer	Advanced gynae or gi cancer Any age, inpatient or outpatient	3 trials: all R, DB, PC [7 uncontrolled pro- and retrospective studies were not included in analysis]	89 in trials	Dexamethasone (2), methylprednisolone (1) at two doses All iv	Non-significant trend for improvement in bowel obstruction with corticosteroids. Treatment may palliate symptoms without affecting morbidity	Strength of evidence limited by small numbers and relevance of outcomes measured	Highlights problems with recruitment. Quality of life should be a primary outcome measure. The longer patients survive, the more significant side effects may become

**McQuay (1999;i3)** Radiotherapy for the palliation of painful bone metastases	Bony metatases from any primary tumour	20 trials: all R, C (blinding often not possible)	3060	External irradiation (6): comparisons of 43 different schedules<br>Radioisotope (8): strontium as mono or combined therapy (6), rhenium (2), samarium (1)	External irradiation and radioisotopes can provide effective analgesia<br>Diverse patients, treatment schedules etc did not permit comparisons between them	Compromises made in analysis to get as much information as possible without creating false validity	Trials heterogeneous: 20 trials yielding 43 different fractionation schedules

R=randomised; DB=double blind; C=controlled; PC=placebo controlled
