# Supplementary material for: How useful are systematic reviews for informing palliative care practice? Survey of 25 Cochrane systematic reviews
Source: BMC Palliat Care. 2008 Aug 20;7:13. doi: 10.1186/1472-684X-7-13 (PMC2532992; doi:10.1186/1472-684X-7-13)
Supplement: Additional file 2 — Adequacy of included studies. Details of design, size, and quality of primary studies in each review. [file 1472-684X-7-13-S2.pdf]

**Additional file 2: Adequacy of included studies**

| Reference | Included studies | Max number of patients | Quality of studies | Exclusion/sensitivity analysis for poor quality |
|---|---|---|---|---|
| **Nicholson (2007;i4)** Methadone for cancer pain | 9 trials: all R, C, and 6 DB | 459 | Oxford Quality Scale<br>QS2=3<br>QS3=2<br>QS4=3<br>QS5=1<br>Allocation Concealment A=3, B=3, C=3 | No |
| **Wiffen (2007;i4)** Oral morphine for cancer pain | 54 trials: all R, C and 37 DB Max 17 trials in any comparison | 3749 | Oxford Quality Scale<br>QS1=7<br>QS2=11<br>QS3=6<br>QS4=18<br>QS5=12 | Sensitivity analysis planned for low quality, but insuff data. |
| **Dewey (2007;i1)** Eicosapentaenoic acid (EPA, an omega-3 fatty acid from fish oils) for the treatment of cancer cachexia | 5 trials: all R, C, 3 DB | 587 | Oxford Quality Scale<br>QS2=1<br>QS3=1<br>QS5=3<br>Allocation Concealment A=1, B=1, C=1 | No |
| **Miles (2006;i4)** Laxatives for the management of constipation in palliative care patients | 4 trials: all R, open | 280 | Oxford Quality Scale and Rinck Scale 1997<br>QS2 + RS3<br>QS3 + RS3.5<br>QS2 + RS2<br>QS3 + RS4<br>Allocation Concealment C=1, D=3 | No |
| **Dennert (2006;i3)** Selenium for alleviating the side effects of chemotherapy, radiotherapy and surgery in cancer patients | 2 trials: both R, C (one presenting preliminary results) | 123 | Oxford Quality Scale<br>QS3=1<br>Other trial pending<br>Allocation Concealment B=1<br>Pending trial Allocation Concealment A=1 | No |
| **Martinez-Zapata (2006;i3)** Calcitonin for metastatic bone pain | 2 trials: both R, C | 90 | Oxford Quality Scale and Oxford Pain Validity Score<br>QS4 + OPVS16<br>QS3 + OPVS13<br>Allocation Concealment both B | No low quality trials |
| **Tsao (2006;i3)** Whole brain radiotherapy for the treatment of multiple brain metastases | 24 trials: various designs, not all full publications | 6353 | Oxford Quality Scale and Detsky score<br>QS1=6<br>QS2=8<br>QS3=6<br>DS 8 to 11=19<br>DS 3=1<br>4 trials not full publications and not scored | Sensitivity analysis for low quality |
| **Ezzo (2006;i2)** Acupuncture-point stimulation for chemotherapy-induced nausea or vomiting | 11 trials: all R, C | 1247 | Table: Randomisation/concealment adequate/sham control/asses'r blind stated/dropouts accounted for<br>Allocation Concealment, A=4, B=3, C=3, D=1 | Sensitivity analysis for allocation concealment and sham vs non sham controls |

| | | | | |
|---|---|---|---|---|
| **Zeppetella (2006;i1)** Opioids for the management of breakthrough (episodic) pain in cancer patients | 4 trials: all R. C | 393 | Oxford Quality Scale<br>QS4- 2<br>QS5- 2<br>Allocation Concealment A=2, B=2 | All trials of high quality |
| **Berenstein (2005;i2)** Megestrol acetate for the treatment of anorexia-cachexia syndrome | 30 trials: all R, C | 4123<br>Only 50% contributed to analysis | Oxford Quality Scale<br>QS3 to 5=20<br>QS<3=10<br>Allocation Concealment A=11, B =19, D=1 | Sensitivity analysis for low quality |
| **Ahmed (200; i1)** Supportive care for patients with gastrointestinal cancer | 4 trials: all R, C | 483 | Oxford Quality Scale and Rinck Scale<br>QS2 + RS5<br>QS3 + RS4.5<br>QS3 + RS5.0<br>QS3 + RS5.5<br>Allocation Concealment A=1, D=3 | Blinding not possible, trials of moderate quality, all included |
| **Ballantyne (2005;i1)** Comparative efficacy of epidural, subarachnoid, and intracerebroventricular opioids in patients with pain due to cancer | No controlled trials<br>72 uncontrolled trials | 2402 | None | No |
| **McNicol (2005;i1)** NSAIDS or paracetamol, alone or combined with opioids, for cancer pain | 42: all R, C | 3084 | Oxford Quality Scale<br>QS1=2<br>QS2=7<br>QS3=22<br>QS4=9<br>QS5=2<br>Allocation Concealment A=1, B=36, D=5 | not used for weighting |
| **Shaw (2005;i1)** Pleurodesis for malignant pleural effusions | 36 trials: all R, all open | 1499 | Cochrane Reviewers Handbook<br>1=5<br>2=1<br>3=5<br>4=11<br>5=10<br>6=1<br>(3 trials not scored)<br>Allocation Concealment A=5, B=31 | No |
| **Fellowes (2004;i3)** Aromatherapy and massage for symptom relief in patients with cancer | 8 trials: all R, C | 357 | Informed by criteria in Oxford Quality Scale. No numerical scoring | No |
| **Quigley (2004;i3)** Opioid switching to improve pain relief and drug tolerability | No RCTs<br>52 reports: case reports (23), retrospective studies/audits (15), prospective uncontrolled studies (14) | Not clear<br>Prospective and retrospective studies ±2000<br>Case reports 55 | No RCTs met inclusion criteria | No |

| | | | | |
|---|---|---|---|---|
| **Jackson (2004;i2)** Drug therapy for delirium in terminally ill patients | 1 trial: R, DB | 30 | Oxford Quality Scale<br>QS5=1<br>Allocation Concealment A=1 | Single trial high quality |
| **Jackson (2004;i1)** Drug therapy for anxiety in palliative care | None | 0 | No RCTs met inclusion criteria | |
| **Roqué (200; i4)** Radioisotopes for metastatic bone pain | 4 trials: all R, DB, PC | 325 | Oxford Quality Scale and Oxford Pain Validity Score<br>QS3 + OPVS5<br>QS5 + OPVS13<br>QS4 + OPVS12<br>QS4 + OPVS15<br>Allocation Concealment A=1, B=3 | Sensitivity analysis for low quality |
| **Bell (2003;i3)** Ketamine as an adjuvant to opioids for cancer pain | 2 trials: both R, DB, cross-over, adjuvant therapy.<br>32 case reports or uncontrolled studies also reviewed | 30 | Oxford Quality Scale and Oxford Pain Validity Score<br>QS3 + OPVS12<br>QS3 + OPVS13<br>Allocation Concealment both B | Yes |
| **Hirst (2002;i4)** Benzodiazepines and related drugs for insomnia in palliative care | None | 0 | No RCTs met inclusion criteria | |
| **Wong (2002;i2)** Bisphosphonates for the relief of pain secondary to bone metastases | 30 trials: all R, 21 DB and PC, 4 open, 5 active (dose response) | 3582 | Oxford Quality Scale<br>QS1=2<br>QS2=8<br>QS3=7<br>QS4=9<br>QS5=4 | Sensitivity analysis for low quality |
| **Jennings (2001;i3)** Opioids for the palliation of breathlessness in terminal illness | 18 trials: all R, DB, PC, cross-over | 292 | Oxford Quality Scale<br>QS3=2<br>QS4-=6<br>Allocation Concealment all B | Trials all adequate quality |
| **Feuer (1999;i3)** Corticosteroids for the resolution of malignant bowel obstruction in advanced gynaecological and gastrointestinal cancer | 3 trials: all R, DB, PC<br>[7 pro- and retrospective studies were uncontrolled and not included in analysis] | 89 in trials | Quality scores (from the NHS criteria)<br>3 included trials: Grade IA<br>7 other studies:<br>IIC = 1<br>IIIC = 6<br>Allocation Concealment A=3 (included trials), D=7 | Sensitivity analysis for missing data, assuming best and worst possible scenarios |
| **McQuay (1999;i3)** Radiotherapy for the palliation of painful bone metastases | 20 trials: all R, C (blinding often not possible) | 3060 | Oxford Quality Scale<br>QS1=2<br>QS2=11<br>QS3=5<br>QS4=1<br>QS5=1<br>Allocation Concealment A=4, B=1, D=15 | Trials all adequate quality, given difficulty of blinding and use of placebo unethical |

R=randomised; DB=double blind; C=controlled; PC=placebo controlled
