# Supplementary material for: How useful are systematic reviews for informing palliative care practice? Survey of 25 Cochrane systematic reviews
Source: BMC Palliat Care. 2008 Aug 20;7:13. doi: 10.1186/1472-684X-7-13 (PMC2532992; doi:10.1186/1472-684X-7-13)
Supplement: Additional file 3 — Quality and validity tools. Details of tools used to assess quality and validity in primary studies. [file 1472-684X-7-13-S3.pdf]

Additional file 3: Quality and validity tools

Tool	Criteria considered
Oxford Quality Score [32]	Randomisation process; blinding process; reporting of withdrawals and dropouts. Max score 5
Oxford Pain Validity Score [33]	Blinding, size, population characteristics, outcome definition, statistical procedures, reporting of withdrawals and dropouts. Max score 16
Rinck [1]	Accrual of study population; patient characteristics; randomisation; attrition and statistical power; definition and execution of interventions; relevance, timing and measurement of outcomes; presentation of results. Max score 7
Detsky [34]	Randomisation process; outcome assessment, inclusion and exclusion criteria; details of intervention, appropriateness of statistics. Max score 15
Juni [35]	Randomisation process; similarity of groups at baseline; blinding; eligibility criteria; provision of care and follow up; reporting of protocol deviations, withdrawals and dropouts; reporting of outcomes, statistical procedures (ITT analysis)
Delphi [36]	Randomisation process; similarity of groups at baseline; blinding; eligibility criteria; provision of care and follow up; reporting of outcomes; ITT analysis
Cochrane Handbook [37]	Similarity of groups at baseline; blinding; eligibility criteria; provision of care and follow up;reporting of protocol deviations, withdrawals and dropouts; reporting of outcomes, statistical procedures. Max score 8
NHS Cancer Services [38]	Uses concept of hierarchy of evidence: Grade I (strong) RCT or review of RCTs; Grade II (fairly strong) prospective study with comparison group; Grade III (weak) retrospective study; Grade IV (weak) cross-sectional study
Ezzo [12]	Randomisation; allocation; use of sham control; outcome assessor blinding; reporting of withdrawals and dropouts
Cochrane Handbook [39]	Allocation concealment: A adequate; B uncertain; C inadequate; D not used
